# Comprehensive analysis of circulating adipokines and hsCRP association with cardiovascular disease risk factors and metabolic syndrome in Arabs

**DOI:** 10.1186/1475-2840-13-76

**Published:** 2014-04-09

**Authors:** Mohamed Abu-Farha, Kazem Behbehani, Naser Elkum

**Affiliations:** 1 Dasman Diabetes Institute, P.O.Box 1180, Dasman 15462, Kuwait

**Keywords:** Adipokine, Arab, Metabolic syndrome, Cardiometabolic risk factors, Lipid profile, hsCRP, Leptin, Adiponectin, Visfatin, Resistin, Adipsin, Low grade inflammation

## Abstract

**Background:**

Cardiovascular diseases (CVD) are a leading cause of death worldwide including the Middle East. This is caused in part by the dysregulation of adipose tissue leading to increased production of pro-inflammatory adipokines and reduction in cardio-protective adipokines such as adiponectin. Ethnicity has been recognized as a major factor in the association between CVD risk factors and the different circulating adipokines. In this study, for the first time, the relationship between traditional cardiovascular risk factors, Metabolic Syndrome (MetS) and circulating level of adipokines in Arab ethnicity was investigated.

**Methods:**

We conducted a population-based cross-sectional survey on 379 adult Arab participants living in Kuwait. Traditional cardiovascular risk factors such as blood pressure (BP), low density lipoprotein (LDL) and triglyceride (TG) were measured. Plasma levels of circulating Leptin, Plasminogen Activator Inhibitor (PAI-1) visfatin, adiponectin, resistin and adipsin were assessed using the multiplexing immunobead-based assay.

**Results:**

Circulating levels of High sensitivity C-Reactive Protein (hsCRP), Leptin, PAI-1 and adiponectin were significantly higher in Arab women than men (p < 0.0001). In multi-variate analysis, the homeostasis model assessment-insulin resistance (HOMA-IR) and body mass index (BMI) showed strong association with most of the biomarkers (p < 0.05). HsCRP showed significant association with all risk factors (p < 0.05). Leptin, PAI-1 and adipsin showed significant positive correlation with BMI, unlike adiponectin which showed inverse correlation (p < 0.05). Subjects in the highest tertile of leptin, PAI-1 and hsCRP had higher odds of having Metabolic Syndrome (MetS) (odd ratio [OR] = 3.02, 95% confidence interval [CI] = 1.47 – 6.19) and (OR = 2.52, 95% CI = 1.45 – 4.35), (OR = 4.26, 95% CI = 2.39 – 7.59) respectively. On the other hand subjects with highest tertile of adiponectin had lower odds of having MetS (OR = 0.22, 95% CI = 0.12 – 0.40). Leptin, PAI-1 and hsCRP showed significant positive association with increased MetS components (P-trend <0.05), while adiponectin was negatively associated with increased MetS components (P-trend <0.0001).

**Conclusion:**

Our results show positive association between hsCRP, leptin, PAI-1 with increased MetS components and increase the odds of having MetS. Adiponectin on the other hand showed inverse correlation with MetS components and associated with reduction in MetS. Overall, our data highlights the significant clinical value these markers have in MetS especially hsCRP which can be used as good marker of low grade inflammation in Arabs.

## Introduction

CVD are one of the leading causes of mortality worldwide [1]. Due to the increase in obesity and MetS, CVD mortality and morbidity are expected to increase even higher posing a huge risk to public health and health care systems around the globe [1]. CVD include cardiomyopathy, cardiac dysrhythmias, myocarditis, myocardial infarction, hypertension and atherosclerosis [2]. A number of risk factors have been identified to be associated with CVD such as hypertension, obesity, smoking, life style, hyperlipidaemia, Type 2 Diabetes (T2D) and MetS [2]. MetS is a cluster of metabolic risk factors that has been shown to cause a two fold increase in cardiovascular outcomes and a 1.5 fold increase in all-cause mortality [3-5]. Central obesity, dyslipidemia, elevated blood pressure; elevated fasting glucose and insulin resistance are the most pivotal components of MetS [6]. MetS is also characterized by a chronic low grade inflammation state which can explain the increased CVD and T2D risk [7]. MetS is also characterized by a low grade inflammation with increased hsCRP level. HsCRP is a marker of low grade inflammation which was shown in many studies to be higher in subjects with MetS and it associates with increased risk of CVD and T2D [7].

The association between MetS, obesity, T2D and CVD is intertwined due to their role in the disruption of homeostasis of important factors such as inflammatory markers and stress related markers in addition to other adipokines produced by adipose tissue [2]. Adipose tissue is an organ that was originally thought to be simply a storage organ for triacylglycerol [8]. Recently, it has been recognized as a metabolically active endocrine organ that affects various biological processes such as energy homeostasis, feeding, immunity and glucose and lipid metabolism amongst others [9]. The main cell types residing in adipose tissues are adipocytes, preadipocytes, fibroblasts, endothelial cells, and immune cells such as macrophages and lymphocytes [9]. Disruption of normal adipose tissue function; as shown with increased obesity; leads to the production and release of proinflammatory, atherogenic, and diabetogenic agents [9]. Consequently, various adipokines such as TNF-α, IL-6, leptin, adiponectin, visfatin, PAI-1 and others are secreted to the blood stream [8,10]. The association between the different adipokines and CVD risk factors has been under investigation to understand their role in cardio-metabolic risk [9]. Adiponectin and PAI-1 for example, are two adipokines with opposing effects on CVD, where adiponectin is thought to be cardio-protective [11] and PAI-1 is atherogenic [12].

Ethnicity has been suggested as a major factor in determining expression level of various metabolic markers and their association with CVD, T2D and MetS [13-17]. Association between adipokines and CVD risk factors and MetS are not very well studied in the Arab population [18]. This prompted our effort to understand the relationship between CVD risk factors and MetS with hsCRP and a group of adipokins in this population. We present population based cross-sectional study in an Arab population living in Kuwait that look at the association between MetS and CVD risk factors such as high fasting blood glucose, high LDL, BMI with a number of adipokines such as leptin, PAI-1 and adiponectin in addition to hsCRP. We also aim to identify markers that can independently associate with MetS and could potentially serve as good prognostic marker for CVD risk in Arab ethnicity.

## Materials and methods

### Study participants

This is a cross-sectional population-based survey undertaken on 379 adult Arab expatriates living in the state of Kuwait. According to the 2011 census, 67.7% of the Kuwait population is expatriates hailing mostly from Arab countries, Indian subcontinent and South East Asia. Subjects originating from Arab countries such as Egypt, Syria, Lebanon, Palestine, Jordan and/or Arab gulf countries were classified as Arabs. Subjects were selected randomly from the computerized register of the Public Authority of Civil Information. Participants with history of CVD were excluded from the study. The study conformed to the principles outlined in the Declaration of Helsinki and was approved by the institutional Ethical Review Committee at Dasman Diabetes Institute. An informed written consent was obtained from all the participants before their enrolment in the study. This study was carried out between June 2011 and August 2012.

### Anthropometric and physical measurements

Physical and anthropometric measurements included body weight, height, waist circumference (WC) as well as systolic blood pressure (SBP) and diastolic blood pressure (DBP). Height and weight were measured, with participants wearing light indoor clothing and bear-footed using calibrated portable electronic weighing scales and portable inflexible height measuring bars. Waist circumference was measured using constant tension tape at the end of a normal expiration, with arms relaxed at the sides, the highest point of the iliac crest and the mid-axillary line. BP was measured with Omron HEM-907XL Digital sphygmomanometer. An average of 3 BP readings, with 5 to 10 minutes rest between each, was obtained. BMI was calculated using the standard BMI formula: body weight (in kilograms) divided by height (in meters squared).

### Laboratory measurements

Blood samples were obtained after an overnight fasting for at least 10 hours and analyzed for fasting glucose, glycated haemoglobin A1c (HbA1c), fasting insulin, and lipid profiles that included TG, total cholesterol (TC), LDL, and high-density lipoprotein (HDL). Glucose and lipid profiles were measured on the Siemens Dimension RXL chemistry analyzer (Diamond Diagnostics, Holliston, MA). HbA1c was determined using the Variant™ device (BioRad, Hercules, CA). All laboratory tests were performed by certified nurses and technicians at the clinical laboratories of DDI, using the Ministry of Health approved methods and quality standards. Insulin resistance was calculated using HOMA-IR formula: fasting blood glucose (FBG) (mmol/L) x fasting Insulin (mU/L) / 22.5.

To measure adipokines, blood was drawn into EDTA tubes. Plasma was obtained after centrifugation, aliquoted and then stored at -80°C. Plasma levels of adipokines were assessed using the multiplexing immunobead array platform (Luminex, Austin, TX). We used the Human Diabetes 10-Plex kit consisting of leptin, PAI-1, resistin, and visfatin) and 2-Plex kit consisting of adiponectin and adipsin (BioRad, Hercules, CA).). Experimental data was processed with Bio-Plex manager software version 6 (Bio-Rad, Hercules, CA) using five-parametric curve fitting. HsCRP secreted level was measured using ELISA kit (Biovendor, USA). All the above mentioned assays were carried out according the manufacturers procedures.

The current recommendations and updated guidelines for the definition, diagnosis and classification of MetS, published by the International Diabetes Federation (IDF), were used [19]. MetS was defined by abdominal obesity and at least two of: fasting blood glucose values ≥ 5.6 mmol/L, hypertension was defined as BP ≥ 130/85 mmHg, under treatment, or a self-report of previously diagnosed hypertension. Hypertriglyceridemia as ≥ 1.7 mmol/L and low HDL cholesterol as < 1.03 mmol/L in men and < 1.29 mmol/L in women. BMI between 18.5 and 24.9 was considered normal, 25 to 29.9, overweight, and equal to or higher than 30, was considered obese. Cutoffs for central obesity were adopted from IDF; they were defined based on race and gender. In our population WC ≥ 94 cm in men and ≥ 80 cm in women was used.

### Statistical analysis

Comparisons between clinical and biochemical profiles between gender were made by Student’s t-test or Wilcoxon test for non-parametric analyses in variables with non-normal distribution. To assess difference in categorical variables between male and female, Chi-Squared test was used. Spearman’s correlation coefficients were estimated to determine associations between adipokine concentrations and anthropometric measurements and biochemical variables. Subjects were classified into tertiles based on their circulating adipokines levels in the overall population. Multivariable logistic regression analysis was performed to estimate odds ratios (ORs) adjusted for covariates and to assess the predictive effect of adipokines on risk for developing MetS. All data are reported as Mean ± Standard Deviation (SD) and range, unless stated otherwise. Research Electronic Data Capture (REDCap) was used for data collections and data management. All statistical assessments were two-sided and considered to be significant when *P*-value < 0.05. All analyses were performed using SAS (version 9.2; SAS Institute, Cary, NC).

## Results

379 subjects (≥20 years of age) were interviewed and included in this analysis, of which 50.9% were female. Women and men had similar mean age (44.1 ± 11.9 years & 44.9 ± 11.5 years respectively). Generally, the men had higher SBP and DBP, FBG, HbA1c, Triglyceride and lower BMI, total cholesterol, HDL, and LDL than women, as shown in Table 1. Overall, women presented with higher circulating levels of hsCRP, leptin, PAI-1, resistin, and adiponectin. There are insignificant difference in visfatin, and adipsin between women and men.

**Table 1:** Clinical and Socio-demographic characteristics of Arab population

**Characteristics**	**All (n = 379) mean ± SD**	**Female (n = 193) mean ± SD**	**Male (n = 186) mean ± SD**	***P*-value**
Age (years)	44.4 ± 11.7	44.1 ± 11.9	44.9 ± 11.5	0.5109
BMI (kg/m^2^)	31.9 ± 6.4	33.2 ± 6.7	30.5 ± 5.8	**<0.0001**
WC (cm)	101.0 ± 14.3	99.3 ± 14.3	102.8 ± 14.1	**0.0153**
SBP (mmHg)	129.9 ± 19.8	124.8 ± 19.6	135.1 ± 18.5	**<0.0001**
DBP (mmHg)	78.3 ± 12.5	76.9 ± 12.6	79.8 ± 12.3	**0.0252**
FBG (mmol/L)	6.2 ± 3.02	5.9 ± 2.5	6.6 ± 3.5	**0.0424**
HbA1c (%)	6.1 ± 1.8	5.7 ± 1.4	6.5 ± 2.0	**<0.0001**
HOMA-IR	3.3 ± 3.9	3.4 ± 4.7	3.1 ± 3.0	0.4486
TC (mmol/L)	5.2 ± 1.1	5.3 ± 1.1	5.1 ± 1.2	**0.0353**
TG (mmol/L)	1.7 ± 1.2	1.5 ± 0.8	1.9 ± 1.4	**0.0045**
HDL cholesterol (mmol/L)	1.1 ± 0.4	1.3 ± 0.4	0.99 ± 0.2	**<0.0001**
LDL cholesterol (mmol/L)	3.3 ± 1.0	3.4 ± 1.0	3.3 ± 1.0	0.2305
Leptin (ng/mL)	4.76(0.20-41.6)	8.35(1.1-41.6)	2.57(0.20-25.6)	**<0.0001**
PAI-1 (ng/mL)	6.87(1.06-29.3)	7.38(1.4-29.3)	5.94(1.06-19.0)	**<0.0001**
Adiponectin (μg/mL)	12.9(1.86-152.1)	15.6(1.86-80.07)	10.6(2.20-152.1)	**<0.0001**
Visfatin (ng/mL)	2.96(0.28-179.2)	3.01(0.30-14.9)	2.9(0.28-179.2)	0.6966
Adipsin (μg/mL)	1.46(0.01-7.5)	1.46(0.48-7.5)	1.46(0.01-5.02)	0.4050
Resistin (ng/mL)	2.82(0.43-13.4)	3.01(0.43-12.6)	2.46(0.56-13.4)	**0.0002**
hsCRP (μg/mL)	2.82(0.01-21.9)	4.53(0.01-21.9)	2.10(0.01-18.2)	**<0.0001**

BMI, body mass index; BP, blood pressure; HDL, high-density lipoprotein; FBG, fasting blood glucose; LDL, low-density lipoprotein.

Results are reported as Mean ± SD except for non-normally distributed metabolic markers that are presented as Median (range).

Age-sex adjusted Spearman partial correlations, Table 2, showed that PAI-1 and adiponectin were significantly associated with most CVD risk factors. HsCRP showed most association as it associated with all risk factors showing strongest association with BMI and WC (R^2^ = 0.50, p < 0.0001) and (R^2^ = 0.46, p < 0.0001) respectively. PAI-1 showed positive correlation with BMI, WC, SBP, DBP in addition to HOMA-IR and TG and negative correlation with HDL (p < 0.05). Resistin and adiponectin on the other hand showed an opposite pattern of correlation. Resistin showed negative correlation with SBP, DBP, FBG and HOMA-IR (p < 0.05). Adiponectin showed negative correlation with BMI, WC, SBP, FBG, HOMA-IR, TG and positive correlation with HDL (p < 0.05). Increased level of visfatin was associated with increased FBG, HOMA-IR, TCHL, LDL, and TG and decreased HDL level (p < 0.05). Similarly plasma leptin level was significantly associated with BMI, WC as well as HOMA-IR (p < 0.0001), Table 3.

**Table 2:** Spearman Correlation (p-value) of Metabolic Markers with cardio-metabolic risk factors

**Markers**	**BMI**	**WC**	**SBP**	**DBP**	**FBG**	**HbA1c**	**HOMA-IR**	**TC**	**HDL**	**LDL**	**TG**
Leptin (ng/ml)	0.48 **(<.0001)**	0.45 **(<.0001)**	0.01 (0.9074)	0.08 (0.1239)	-0.02 (0.6386)	-0.1 (0.0614)	0.32 **(<.0001)**	0.02 (0.7071)	-0.01 (0.8035)	0.02 (0.7113)	0.10 (0.0687)
Pai_1 (ng/ml)	0.20 **(0.0002)**	0.14 **(0.0066)**	0.11 **(0.0318)**	0.10 **(0.0491)**	0.06 (0.2188)	-0.1 (0.0872)	0.20 **(0.0002)**	0.03 (0.5377)	-0.18 **(0.0009)**	0.05 (0.3823)	0.21 **(<.0001)**
Adiponectin (μg/ml)	-0.26 **(<.0001)**	-0.21 **(<.0001)**	-0.13 **(0.0165)**	-0.17 (0.2107)	-0.22 **(<.0001)**	-0.18 **(0.0007)**	-0.34 **(<.0001)**	-0.08 (0.1212)	0.33 **(<.0001)**	-0.10 (0.0509)	-0.28 **(<.0001)**
Visfatin (ng/ml)	0.10 (0.0663)	0.02 (0.7477)	0.01 (0.8272)	-0.01 (0.9019)	0.17 **(0.0010)**	-0.01 (0.8821)	0.20 **(0.0001)**	0.13 **(0.0138)**	-0.13 **(0.0184)**	0.13 **(0.0179)**	0.12 **(0.0195)**
Adpisin (ng/ml)	0.33 **(<.0001)**	0.3 **(<.0001)**	0.03 (0.6119)	0.10 (0.2710)	-0.12 **(0.0207)**	-0.15 **(0.0048)**	0.02 (0.6884)	-0.08 (0.1235)	-0.05 (0.2055)	-0.10 (0.0523)	0.08 (0.1175)
Resistin (ng/ml)	-0.07 (0.1751)	-0.06 (0.2596)	-0.11 **(0.0448)**	-0.14 **(0.0082)**	-0.14 **(0.0074)**	-0.09 (0.0776)	-0.14 **(0.0006)**	-0.06 (0.28)	-0.05 (0.3937)	-0.04 (0.4869)	-0.01 (0.8508)
hsCRP (μg/ml)	0.50 **(<0.0001)**	0.46 (<**0.0001)**	0.14 **(0.0085)**	0.16 **(0.0029)**	0.13 **(0.0148)**	0.19 **(0.0004)**	0.36 **(<0.0001)**	0.15 **(0.0055)**	-0.18 **(0.0008)**	0.15 **(0.0050)**	0.15 **(0.0046)**

Values are age-sex adjusted Sperman partial correlation coefficients and *P* values for correlations of metabolic markers and cardiovascular disease risk factors.

**Table 3:** Multiple logistic regression models for MetS in relation to metabolic markers

**Biomarkers**	**T1**	**T2**	**T3**	***P*-trend**
	**Reference**	**OR (95% CI)**	**OR (95% CI)**	
Adiponectin (μg/ml)	1	0.38 (0.22 – 0.67)	0.22 (0.12 – 0.40)	<0.0001
Leptin (ng/ml)	1	2.71 (1.46 – 5.02)	3.02 (1.47 – 6.19)	0.0029
PAI-1 (ng/ml)	1	1.81 (1.06 – 3.09)	2.52 (1.45 – 4.35)	0.0037
Visfatin (ng/ml)	1	1.01 (0.60-1.70)	1.13 (0.66-1.92)	0.8860
Adipsin (μg/ml)	1	1.17 (0.69-1.97)	1.48 (0.87-2.54)	0.3528
Resistin (ng/ml)	1	1.48 (0.86-2.55)	1.06 (0.62-1.81)	0.3015
hsCRP (μg/ml)	1	3.21 (1.85-5.58)	4.26 (2.39-7.59)	<0.0001

The MetS models adjusted for age, and gender.

Tertile values are expressed as:

Adiponectin: T1 (<10.0; n = 127), T2 (10.0 – 16.41; n = 127), and T3 (>16.41; n = 125); Leptin: T1 (<3.27; n = 128), T2 (3.27 – 7.23; n = 126), and T3 (>7.23; n = 125); Pai-1: T1 (<5.58; n = 129), T2 (5.58 – 8.00; n = 126), and T3 (>8.00; n = 124); Visfatin: T1 (2.43; n = 129), T2 (2.43 – 3.79; n = 126), and T3 (>3.79; n = 124); Adipsin: T1 < (1.28; n = 127), T2 (1.28 – 1.65; n = 126), and T3 (>1.65; n = 126). Resistin: T1 (<2.35; n = 129), T2(2.35 – 3.40; n = 125), and T3 (>3.40; n = 125); hsCRP: T1 (<1.71; n = 130), T2 (1.71 – 5.12; n = 125), T3 (>5.12; n = 124).

**Figure 1:**
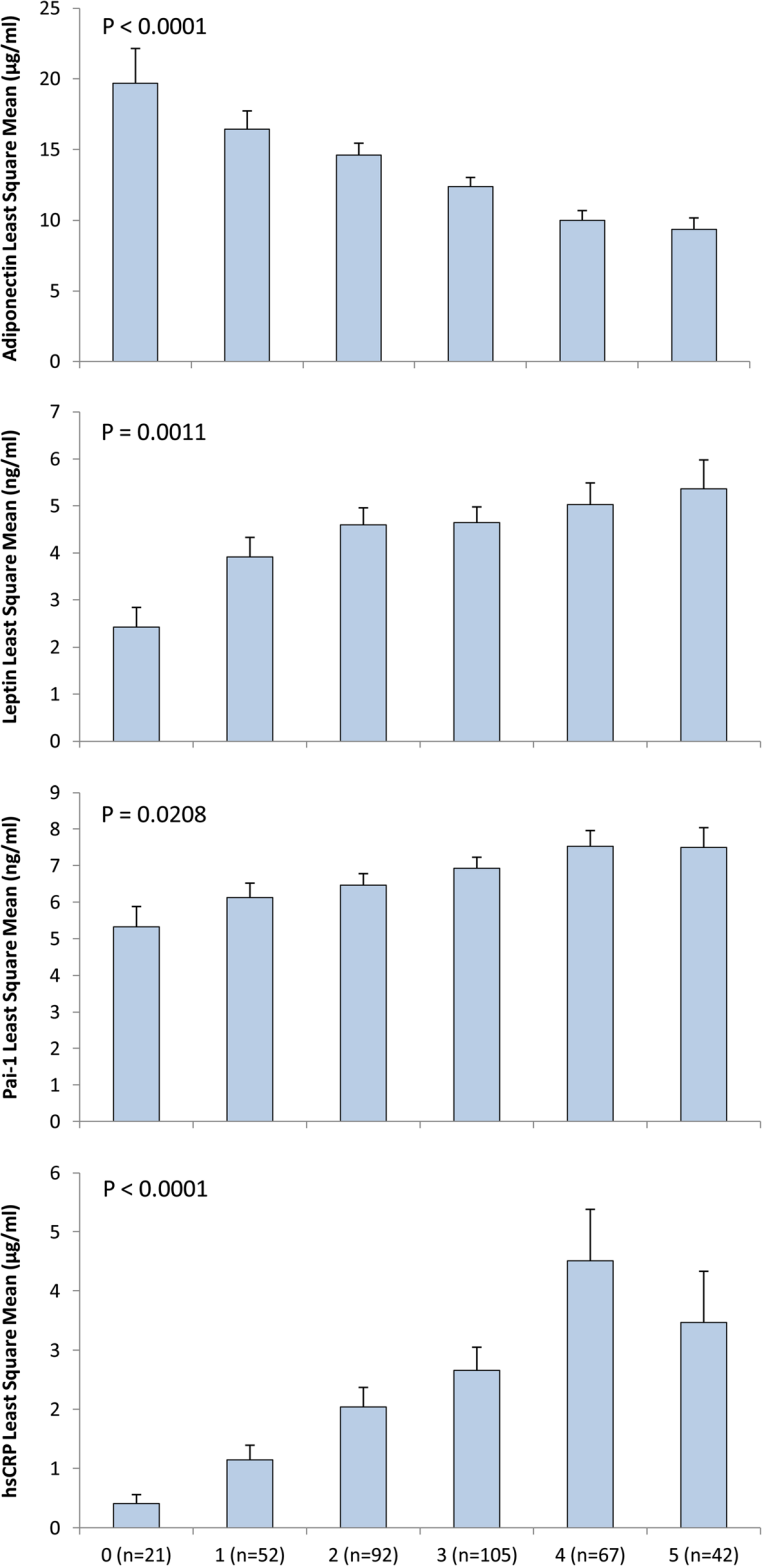
Age-gender adjusted least square means of concentrations of adiponectin, leptin, and PAI-1 and hsCRP according to the number of MetS components.

After adjusting for age and gender subjects in the highest tertile of leptin, PAI-1and hsCRP had higher odds of having MetS (OR = 3.02, 95% CI = 1.47 – 6.19) (*P*-trend = 0.0029), (OR = 2.52, 95% CI = 1.45 – 4.35) (*P*-trend = 0.0037) (OR = 4.26, 95% CI = 2.39 – 7.59) (*P*-trend < 0.0001) respectively. Subjects in the highest tertile of adiponectin had lower odds of having MetS (OR = 0.22, 95% CI = 0.12 – 0.40) (*P*-trend < 0.0001). On the other hand, subjects in the highest tertiles of resistin, adipsin and visfatin did not show any significant association with MetS (Table 3). Age-gender adjusted least square means of leptin, PAI-1, hsCRP and adiponectin concentrations showed significant association with increasing number of MetS components (Figure 1). Increased leptin, PAI-1 and hsCRP plasma level was directly associated with increased number of MetS (*P*-trend < 0.05). Adiponectin showed opposite effects as it was inversely associated to increased number of MetS in this population (*P*-trend < 0.0001).

## Discussion

The main objective of the current study was to identify metabolic markers that are associated with traditional cardiovascular disease risk factors and MetS in Arab population. Our study showed sexual dimorphism in plasma level of leptin, PAI-1, hsCRP, resistin and adiponectin with women showing higher levels of these metabolic markers in Arabs. hsCRP showed significant positive correlation with all risk factors except for HDL where it was inversely associated. Leptin, PAI-1 and adipsin showed positive correlation with BMI. Visfatin level was not associated BMI level unlike other adipokines. Adiponectin showed highest association with CVD risk factors as it negatively associated with age, BMI, sex, SBP, HOMA-IR and LDL. It was also negatively associated with increased odds of MetS and increasing MetS components. Lepetin and PAI on the other hand were associated with higher odds of MetS and with increasing number of MetS components. Taken together, this data highlights some of the potential role these metabolic markers play in development of MetS and CVD as well as their diagnostic value in this population.

### HsCRP, PAI-1 and Leptin Association with Metabolic Risk Factors and MetS

MetS is defined by different health organizations as a cluster of metabolic risk factors that constitute an increased risk for developing T2D and CVD [20][21]. Central obesity, dislypidemia hypertension and hyperglycemia are key components in the definition of MetS [21,22]. MetS is also characterized by a chronic low grade inflammation condition [20,23]. Current definitions of MetS lack a component that measures inflammation status. HsCRP has been suggested as a sensitive marker that is predictive of MetS as well as development of cardiovascular problems [20,22-24]. Our data showed that hsCRP correlated with all the measured risk factors and was most predictive of the development of MetS in this population. HsCRP strong association with increased number of MetS components highlights its beneficial use as prognostic marker for metabolic disorders in this population as well.

Using a wide range of adipokines our current study showed that women possessed higher circulating levels of leptin, PAI-1 and adiponectin. Even though all of these markers showed a significant difference, it was highest for leptin at almost three folds followed by adiponectin at 1.6 fold difference. This difference in leptin level for example is consistent with levels reported in other studies such as Zuo et al., reported that men had 1.45 ng/ml compared to 8.32 ng/ml in women [25]. This difference can be explained by the difference in sex hormones especially in women as a result of menopause which reduces estrogen production a factor that has been shown to lead to obesity in women [26]. Consistent with data reported in literature for other populations, leptin showed positive association with BMI and HOMA-IR.

PAI-1 and adiponectin were remarkably associated with seven and eight of the studied risk factors respectively. PAI-1 is a serine protease inhibitor that plays an important role in fibrinolysis due to its inhibition of plasminogen activation [27]. Elevated blood levels of PAI-1 have been linked to high reoccurrence events of myocardial infarction ([28-30]), Atherosclerosis [31] and MetS. The positive association of PAI-1 with all of the risk factors, except for HDL, is in agreement with literature [12]. In accordance to our data, PAI-1 has been shown to positively correlate with atherosclerosis risk factors such as obesity, hyperinsulinemia and hypertriglyceridemia [32,33]. In general, PAI-1 positively associates with MetS and its components in our study. Other studies have shown that PAI-1 level are reduced in response to interventional therapies that leads to reduction in MetS components such as insulin resistance or weight reduction [34]. Thus, the predictive ability of PAI-1 for CVD diminishes after adjusting for MetS components. This suggests that MetS is a prerequisite to high PAI-1 blood circulating level [12]. It can also explain the lack of association with as many markers in the multivariate analysis in our population.

### Adiponectin, a cardio-protective adipokine

Adiponectin on the other hand associated with most CVD risk factors at the univariate and multivariate level. It associated with six of the risk factors showing the strength of this marker in predicting CVD risk. We also showed that decreasing concentration of adiponectin associated with an increasing number of CVD risk factors particularly in women highlighting the gender difference. The importance of adiponectin in CVD is well documented as its plasma level has been shown to be attenuated by cardiac pathologies such as coronary artery disease [35,36], hypertension [37] and myocardial infarction [38]. Further, low plasma levels of adiponectin are associated with obesity, T2D and MetS which are main CVD risk factors [39]. Adiponectin is believed to be a cardio-protective cytokine due to its insulin sensitizing, anti-inflammatory and antiatherogenic properties [11,40]. In Human, a reported single nucleotide polymorphism is associated with a reduction in adiponectin plasma levels leaving individuals with these mutations predisposed to insulin resistance [41]. Iindividuals carrying this mutation were highly susceptible to hypertension and coronary artery disease supporting the protective role this protein plays [42].

### Adipsin, visfatin and resistin association with metabolic markers

Our data sheds light on the association between visfatin and adipsin with CVD risk factors in Arab population. No independent association between BMI and visfatin was observed. Association between obesity and visfatin has been contested with some studies showing positive correlation with obesity [43] while others failed to show any correlation [44]. One of the factors suggested for this discrepancy was ethnicity [45]. The fact that we show no association between visfatin and BMI emphasizes the importance of ethnic variations and establishing the association between the different biomarkers in different ethnicities.

Adipsin on the other hand is a serine protease that has been shown to be upregulated in obesity. It is involved in triglyceride metabolism through the cleavage of complement factor C3 to C3a that stimulates TG production in adipose tissue [46]. Maresh et al. showed that adipsin was upregulated in a type 1 diabetes model possibly to compensate for the increased fat utilization in insulin-deficient animals [46]. Our data shows a trend of increased adipsin production in subjects with higher BMI and WC that is consistent with the known function of adipsin.

Resistin is another adipokines that was found to be increased in a diet induced obesity mouse model, as well as genetically modified diabetic and obese mouse models [47]. Resistin was also found to be a mediator of insulin resistance in rodents. Unlike rodents, resistin was predominantly expressed in monocytes and macrophages [47]. Even though, resistin was associated with obesity and insulin resistance in rodents, human data is conflicting in this regard. Some studies show association with obesity and insulin resistance while others do not show any association or negative association [47-49]. Our data shows negative association between resistin and HOMA-IR, highlighting the importance of ethnic studies to better understand the function of these metabolic markers.

### Ethnicity and metabolic markers

Difference in the level of various metabolic markers between different ethnic groups is well documented and is suggested as a main reason for the discrepancies reported in different studies [14-17,50-52]. For example, Caucasians have a higher level of adiponectin than other ethnicities such as Asians or African Americans [13,53]. In a multiethnic study, Morimoto et al. reported significant ethnic differences in the level of leptin, adiponectin, IL-6 and CRP between Caucasians, Japanese Americans, Latinos, African Americans and Native Hawaiians [17]. TNF-α on the other hand was the only biomarker that did not show any significant difference [17]. This ethnic difference in the plasma level of metabolic markers is also reflected on the association between these markers and various risk factors [16,45]. For example, Sulistyoningrum et al. showed that decrease in adiponectin level was associated with greater increase in insulin resistance as measured by HOMA-IR in Aboriginals, Chinese, and South Asians compared to Europeans [16].

Available data regarding the level of metabolic markers in the Arab population are scarce. Therefore, this study represents an important milestone in understanding the role of these metabolic markers in Arabs. On the other hand, studies linking various metabolic markers to the genetic background and comparing Arabs to other ethnicities are also scarce. However; Genetic variation in adiponectin and its association with metabolic disorders is one of the well studied markers in Arabs [54-57]. For example, Mtiraoui et al. reported association between adiponectin single nucleotide polymorphisms and T2D in Tunisian Arabs [56]. In another study, Zadjali et al. showed that rs266729 in the adiponectin gene was associated with traits defining obesity in Arab population [54]. As a result, such studies looking at the association between metabolic markers genetic variants and metabolic disorders in Arabs compared to other ethnicities will be crucial to better understand the ethnic variation in this ethnicity.

### Study strengths and limitations

The strength of the current study is the fact that it comprehensively investigates the correlation between the CVD risk factors and adipokines in Arab population. It also highlights the importance of hsCRP in predicting MetS and potential use as a prognostic marker for increased risk of T2D and CVD. Nonetheless, we have a few limitations in this study that can be overcome in future studies. The first one is the lack of comparison with other populations especially Caucasians to establish the level of adipokines in Arabs to a well studied population. Establishing such a cohort will be a difficult task to do in Kuwait where percentage of Caucasians is limited. The second limitation regarding the study design, the cross-sectional design used here makes it impossible to determine any causal relationship between circulation levels of adipokines and CVD. It would also be important to study the effect of nutrients in future studies as they have been shown to modulate adipokines as recently shown by Juanola-Falgarona et al. [58].

## Conclusions

In conclusion, this study is one of the first studies to investigate the association of metabolic biomarkers level with CVD risk factors and MetS in Arab population. Our data demonstrated the positive association between hsCRP, leptin and PAI-1 with increased number of CVD risk factors, MetS and MetS components. We also showed that adipsin was positively correlating with BMI while visfatin showed no significant correlation with BMI; both proteins did not correlate with MetS components in this population. On the other hand, adiponectin was negatively associated with many of the CVD risk factors and MetS components showing its beneficial role in this population as observed in other populations. In conclusion, hsCRP, leptin, PAI-1 and adiponectin show strongest association with CVD risk factors and MetS in Arabs. Our findings also emphasize the use of hsCRP as an important measure of low grade inflammation and its association with MetS.

## Abbreviations

LDL: Low Density Lipoprotein; HDL: High Density LipoproteinTG, Triglyceride; TC: Total Cholesterol; SBP: Systolic Blood Pressure; DBP: Diastolic Blood Pressure; BMI: Body Mass Index; WC: Waist Circumference; FBG: Fasting Blood Glucose; HOMA-IR: Homeostasis Model Assessment-Insulin Resistance; HbA1C: glycated Haemoglobin A1c; CVD: Cardiovascular Diseases; T2D: Type 2 Diabetes; hsCRP: High sensitivity C-Reactive Protein; PAI-1: Plasminogen Activator Inhibitor; MetS: Metabolic Syndrome; OR: Odd Ratio; CI: Confidence Interval.

## Competing interests

The authors declare that they have no competing interests.

## Authors’ contributions

MA: Data analysis and wrote the manuscript. KB: Conception of the study. NE: Conception & design of the study, handled data analysis and interpretation, and wrote the manuscript. All authors read and approved the final manuscript.
